# Vitamin D deficiency in pregnancy is not associated with diabetes mellitus development in pregnant women at low risk for gestational diabetes

**DOI:** 10.4274/tjod.10170

**Published:** 2016-03-10

**Authors:** Mehmet Bal, Gülçin Şahin Ersoy, Ömer Demirtaş, Sefa Kurt, Abdullah Taşyurt

**Affiliations:** 1 Tepecik Education and Research Hospital, Department of Obstetrics and Gynecology, İzmir, Turkey

**Keywords:** Gestational diabetes mellitus, 25 (OH) vitamin D3, Vitamin D deficiency

## Abstract

**Objective:**

We aimed to investigate the effect of vitamin D deficiency as a risk factor for the development of gestational diabetes mellitus (GDM) among pregnant women without known risk factors.

**Materials and Methods:**

The study was conducted on pregnant women who had been under regular follow-up and had low risk for GDM development. The patients were divided into two groups according to the presence of GDM; GDM and no GDM (control) group. Body mass index (BMI), sociodemographic data including level of education and nutritional habits were recorded. Serum 25 (OH) vitamin D_3_ levels, hemoglobin, hematocrit, and mean corpuscular volume (MCV) values were measured. An oral glucose tolerance test was performed, between 24 and 28 weeks of pregnancy.

**Results:**

GDM ratio was calculated as 4.6%. The false positive rate of 50 g oral glucose load screening test was found to be 16.5%. The BMI levels of women diagnosed as having GDM and those with no GDM group at the beginningof the pregnancy period were calculated as 24.3±2.6 and 22.8±1.6 kg/m2 respectively, exhibiting a statistically significant difference between the two groups (p=0.001). Hemoglobin, hematocrit, and MCV values did not show a statistically significant difference between the two groups (p>0.05). The levels of 25 (OH) vitamin D_3_ of the study groups were found comparable in both groups (p=0.13).

**Conclusion:**

Plasma levels of vitamin D may not be a contributing factor for the development of GDM in women with a low risk for GDM.

## PRECIS:

We evaluated the effect of vitamin D deficiency as a contributing factor for the development of gestational diabetes among pregnant women who have a low risk for gestational diabetes

## INTRODUCTION

Gestational diabetes mellitus (GDM) is defined as carbohydrate intolerance that appears after 24 weeks of gestation^(1,2)^. There is increasing evidence that vitamin D, a secosteroid synthesized in the skin, plays a vital role in the maintenance of normal glucose balance. 1, 25-dihydroxy vitamin D_3_ is responsible for the increase of the production and secretion of insulin and for the concurrent decrease of insulin resistance^(3,4)^.

The high prevalence of vitamin D deficiency among pregnant women is a well established. However, scientific data regarding the correlation of vitamin D with glucose balance during pregnancy and the occurrence of GDM is still insufficient and inconsistent^(5)^. In this study, we aimed to determine whether vitamin D levels in maternal plasma were a risk factor for the development of GDM among pregnant women who had no other known risk factors for GDM.

## MATERIALS AND METHODS

This cross-sectional study was conducted on pregnant women in the first trimester who were receiving antenatal care in the Aegean Obstetrics and Gynecology Education and Research Hospital in İzmir, Turkey. The study was approved by the local ethics committee of the hospital, and written consent was given by each patient. All subjects had low risk for GDM development without any other accompanying medical condition. We accepted the risk factors for GDM development as those determined by the American Diabetes Association (ADA) and the American Congress of Obstetricians and Gynecologists (ACOG)^(6,7)^. Therefore, women with obesity, aged more than 35 years, with prior history of gestational diabetes mellitus in a previous pregnancy, and family history of diabetes mellitus were excluded. The inclusion criteria for the study were pregnant women aged between 15 and 35 years; a negative C-reactive protein value; the absence of accompanying illness that could interfere with the interpretation of diabetes (e.g. Cushing’s syndrome, acromegaly); a medical history devoid of alcohol consumption; diabetogenic agent use (e.g. steroids); multiple pregnancy; hematological illness; and the absence of glucose in urine analysis.

One thousand four hundred pregnant women with a low risk of gestational diabetes who applied to our clinic in the first trimester of pregnancy were enrolled in the study. Sociodemographic variables, hemoglobin, hematocrit, and mean corpuscular volume (MCV) levels were all recorded. A 50-g oral glucose load screening test was conducted between the 24^th^ and 28^th^ weeks of gestation. In accordance with the guidelines recommended by the ACOG, the two-stage approach was used for the screening and diagnosis of GDM. All pregnant women with a low risk for GDM ingested a solution containing 50 g of sugar and venous blood samples were collected one hour later; patients whose plasma glucose levels were above 140 mg/dL were considered as abnormal. Those with abnormal results underwent a 100-g oral glucose tolerance test (OGTT) approximately 2 weeks later. Women who matched at least two of the criteria listed below were diagnosed as having GDM:

Fasting blood glucose level ≥95 mg/dL (5.3 Mmol/L),

Blood glucose level at the 1^st^ hour ≥180 mg/dL (10 Mmol/L),

Blood glucose level at the 2^nd^ hour ≥155 mg/dL (8.6 Mmol/L),

Blood glucose level at the 3^rd^ hour ≥140 mg/dL (7.8 Mmol/L).

Of the 1322 patients with normal test results, 50 women were randomly selected as the control group using a simple computerized randomization chart generator. Seventy-eight patients with abnormal test results proceeded with the 100-g OGTT and 65 pregnant women were diagnosed as having GDM. Fifteen patients who did not comply with the regular follow-ups, who had an obstetric complication, and those who declared their intention to leave the research protocol were excluded from the study. Following the OGTT examination, 25 (OH) vitamin D_3_ levels were measured and recorded for each patient, which is accepted as the most dependable form of vitamin D in reflecting the actual level of vitamin D in the body. All blood sampling was undertaken in the summer season because vitamin D levels show seasonal variation. In both groups, the measurement of serum 25 (OH) vitamin D_3_ levels was performed using liquid-liquid extraction with a liquid UPLC/MS/MS chromatography-tandem mass spectrometer (manufacturer required). The lowest level of 25 (OH) vitamin D_3_ without any change in serum PTH levels is 30 ng/mL, thus this was accepted as the lower limit for vitamin D adequacy^(8,9,10)^. Levels between 20 and 30 ng/mL were designated as borderline deficiency, and below 20 ng/mL was overt deficiency.

### Statistical Analysis

The arithmetic mean, standard deviation, frequency and ratio values were used for the descriptive statistical analysis of the data. The distribution of data was checked using the Kolmogorov-Simirnov test. Normally distributed data were analyzed using the independent sample t-test, data without normal distribution were evaluated using the Mann-Whitney U test. Nominal data were analyzed using the Chi-square test.

## RESULTS

Following the 100-g OGTT, 65 out of the 78 pregnant women with an abnormal 50-g glucose screening test were diagnosed as having GDM as per the ACOG criteria (the false positive rate of the 50-g OGT was found 16.5%). The GDM ratio was calculated as 4.6%. Fifty of the 65 pregnant women with GDM met the inclusion criteria. The BMI of the women who were diagnosed as having GDM was calculated as 24.3, whereas it was 22.8 in the control group; there was a statistically significant difference between the two groups (p=0.001). The demographic features of both groups are summarized in Table 1.

The levels of hemoglobin, hemotocrit, and MCV were similar in both groups (p>0.05). The level of 25 (OH) vitamin D_3_ in patients did not exhibit a statistically significant difference between the two groups (p=0.13). Moreover, the comparison of fish and milk consumption as well as the rate of vitamin pills use did not reveal a statistically significant difference between the groups (p>0.05) (Table 1). In addition, our study population lived in a sunny region and all patients had normal sun exposure frequency (≥ 3day/week and ≥30 minutes/day) and none were wearing strict religious clothing.

## DISCUSSION

The aim of the study was to investigate the association between vitamin D deficiency and GDM development in pregnant women at low risk for GDM. Our results showed that vitamin D levels were not associated with the development of GDM. To the best of our knowledge, this is the first study to evaluate the effects of vitamin D levels on the development of GDM in pregnant women at low risk for GDM.

GDM is defined as the intolerance for carbohydrates starting from the 24^th^ week of gestation^(1,2)^. Factors that cause insulin resistance have always attracted the attention of scientists; the recent discovery of the interrelations between vitamin D and the cardiovascular system, immunomodulation, metabolic syndrome, autoimmunity and certain carcinomas has enhanced the popularity of vitamin D. Some studies detected that pancreatic β cells express a vitamin D receptor, and allelic variations in genes involved in vitamin D metabolism suggested a role for 25 (OH) vitamin D_3_ in the regulation of insulin secretion and glucose intolerance^(11)^. In addition, it has been shown that vitamin D deficiency in experimental animals impaired insulin release and glucose tolerance^(12,13)^. Although deficiency of 25 (OH) vitamin D_3_ levels has long been suspected as a risk factor for insulin resistance in GDM pregnancies, the association of vitamin D and GDM is still controversial. Similarly, it has been reported that increased serum iron levels might be related to GDM risk in pregnant women^(14,15)^. Also, a study conducted by Afkhami-Ardekani and Rashidi^(16)^ showed that women with GDM had significantly higher levels of serum ferritin, iron, hemoglobin and MCV. In order to evaluate this factor, we also examined the MCV value, which is a robust index of iron status in pregnant women^(17)^. The comparison of the MCV levels revealed no statistically significant difference between groups.

The ADA reported the prevalence of GDM in pregnant women as 4%^(6)^. In Turkey, the prevalence of GDM varies between 3-8%^(18)^. In the present study, the GDM ratio was found as 4.6%; however, all women in the study were at low risk for GDM development, thus the GDM ratio in this study does not reflect the actual incidence of GDM in Turkey.

Several previous studies demonstrated results consistent with our findings; Flood-Nichols et al.^(19)^, Makgoba et al.,^(20)^ Farrant et al.,^(21)^ and Park et al.,^(22)^ reported no association in the United States of America, the United Kingdom, India, and Korea between vitamin D deficiency and development of GDM. However, Lacroix found that maternal vitamin D deficiency was a risk factor for developing GDM in pregnant women in Canada^(23)^. These studies did not evaluate the effect of vitamin D deficiency in pregnant women who were at lower risk for developing GDM. Therefore, in the studies that found a higher correlation between vitamin D deficiency and GDM, the results may have been related with other predisposing factors such as older maternal age and prior history of gestational diabetes in a previous pregnancy. In our study, these factors were excluded, thus we suggest that lower vitamin D levels had no association with development of GDM in Turkish pregnant women at low risk for gestational diabetes. Even though there is no consensus about the prophylactic vitamin D treatment for the prevention of developing GDM in higher risk women, vitamin D supplementation is essential for pregnant women in terms of its potentially beneficial effects. It is known that vitamin D supplementation therapy reduces the incidence of preterm labor, gestational diabetes, and small-for-gestational-age babies^(24)^. There are some limitations of the study; first, the sample size was rather small because it had been designed as a prospective study. The total number of pregnant women was 1400 but only 65 developed GDM; this low ratio of GDM (+) women in the study group was thought to be caused by the inclusion of women with lower risk of GDM to the study. Secondly, we did not measure vitamin D levels in the first trimester; therefore, the influence of the variation in vitamin D levels was not evaluated.

## CONCLUSION

The physiologic properties of the pregnancy period differ from normal female physiology and there are many factors such as pregnancy hormones that act on the emergence of insulin resistance during the physiologic development of pregnancy. Therefore, we were unable to find a direct correlation between vitamin D levels and GDM development as documented in several previous studies. New and long-term prospective studies in a broader patient series including pregnant women are needed to further clarify the precise relationship between vitamin D and GDM.

## Figures and Tables

**Table 1:**
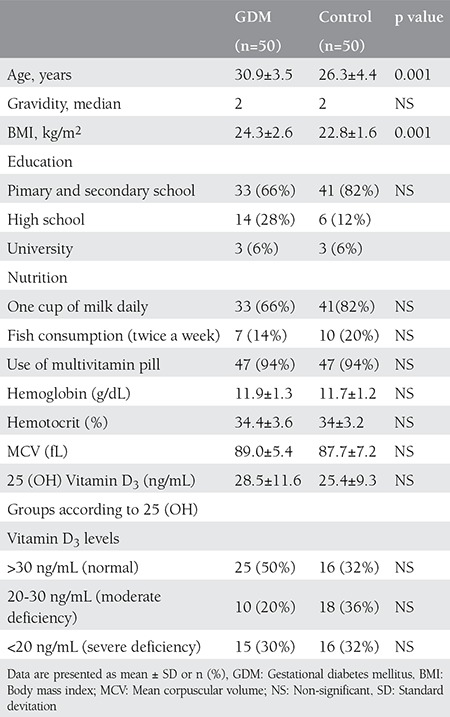
Patient characteristics and 25 (OH) vitamin D_3_ levels
